# Maternal History of Adverse Experiences and Posttraumatic Stress Disorder Symptoms Impact Toddlers’ Early Socioemotional Wellbeing: The Benefits of Infant Mental Health-Home Visiting

**DOI:** 10.3389/fpsyg.2021.792989

**Published:** 2022-01-17

**Authors:** Julie Ribaudo, Jamie M. Lawler, Jennifer M. Jester, Jessica Riggs, Nora L. Erickson, Ann M. Stacks, Holly Brophy-Herb, Maria Muzik, Katherine L. Rosenblum

**Affiliations:** ^1^School of Social Work, University of Michigan, Ann Arbor, MI, United States; ^2^School of Social Work, Wayne State University, Detroit, MI, United States; ^3^Department of Psychology, Eastern Michigan University, Ypsilanti, MI, United States; ^4^Department of Psychiatry, University of Michigan, Ann Arbor, MI, United States; ^5^Mother Baby Program, Department of Psychiatry, Hennepin Healthcare, Minneapolis, MN, United States; ^6^Merrill Palmer Skillman Institute, Wayne State University, Detroit, MI, United States; ^7^Department of Human Development and Family Studies, Michigan State University, East Lansing, MI, United States; ^8^Department of Obstetrics and Gynecology, University of Michigan, Ann Arbor, MI, United States

**Keywords:** infant mental health, parent-infant psychotherapy, maternal PTSD, Infant Mental Health-Home Visiting, infant socioemotional development, maternal childhood adversity, toddler socioemotional development

## Abstract

**Background:**

The present study examined the efficacy of the Michigan Model of Infant Mental Health-Home Visiting (IMH-HV) infant mental health treatment to promote the socioemotional wellbeing of infants and young children. Science illuminates the role of parental “co-regulation” of infant emotion as a pathway to young children’s capacity for self-regulation. The synchrony of parent–infant interaction begins to shape the infant’s own nascent regulatory capacities. Parents with a history of childhood adversity, such as maltreatment or witnessing family violence, and who struggle with symptoms of post-traumatic stress may have greater challenges in co-regulating their infant, thus increasing the risk of their children exhibiting social and emotional problems such as anxiety, aggression, and depression. Early intervention that targets the infant–parent relationship may help buffer the effect of parental risk on child outcomes.

**Methods:**

Participants were 58 mother–infant/toddler dyads enrolled in a longitudinal randomized control trial testing the efficacy of the relationship-based IMH-HV treatment model. Families were eligible based on child age (<24 months at enrollment) and endorsement of at least two of four socio-demographic factors commonly endorsed in community mental health settings: elevated depression symptoms, three or more Adverse Childhood Experiences (ACEs) parenting stress, and/or child behavior or development concerns. This study included dyads whose children were born at the time of study enrollment and completed 12-month post-baseline follow-up visits. Parents reported on their own history of ACEs and current posttraumatic stress disorder (PTSD) symptoms, as well as their toddler’s socioemotional development (e.g., empathy, prosocial skills, aggression, anxiety, prolonged tantrums).

**Results:**

Maternal ACEs predicted more toddler emotional problems through their effect on maternal PTSD symptoms. Parents who received IMH-HV treatment reported more positive toddler socioemotional wellbeing at follow-up relative to the control condition. The most positive socioemotional outcomes were for toddlers of mothers with low to moderate PTSD symptoms who received IMH-HV treatment.

**Conclusion:**

Results indicate the efficacy of IMH-HV services in promoting more optimal child socioemotional wellbeing even when mothers reported mild to moderate PTSD symptoms. Results also highlight the need to assess parental trauma when infants and young children present with socioemotional difficulties.

## Introduction

Science illuminates the role of parental “co-regulation” of infant emotion as a pathway to young children’s capacity for socioemotional wellbeing, including the ability to express and manage emotions, and manage attention and impulses that facilitate social relationships (Geva and Feldman, 2008). Parents with a history of childhood adversity, such as maltreatment or witnessing family violence, may have greater challenges in co-regulating their infant, thus increasing the risk of their children exhibiting social and emotional problems such as anxiety, aggression, and depression (Ahlfs-Dunn and Huth-Bocks, 2014; Steele et al., 2016). In this paper, we summarize the relationship between maternal psychological wellbeing and infant socioemotional development. Specifically, we examine the influence of maternal early adversity and current symptoms of posttraumatic stress on infant socioemotional wellbeing. Finally, we explore the efficacy of Infant Mental Health-Home Visiting (IMH-HV) (Tableman and Ludtke, 2020) in promoting the socioemotional wellbeing of infants whose mother has a history of early adversity and current symptoms of posttraumatic stress disorder (PTSD).

### Infant Socioemotional Development

Emotion regulation is an important aspect of infant socioemotional wellbeing. It acts to reduce negative emotions and, importantly, also serves in “amplification, an intensification of positive emotion, a condition necessary for more complex self-organization” (Schore, 2001b, p. 21). The neuroscience of the development of early childhood emotion regulation largely focuses on parent–child interactions and the parent–infant attachment system. Exchanges between parent and child in the form of emotional or affective communication, sharing emotion, and the caregiver’s “empathic understanding” of the child’s emotional state are important pathways in the process of emotion regulation (Schore, 2001a; Trevarthen, 2001; Manian and Bornstein, 2009). Ultimately, the synchrony children experience in infancy primes them for emotion regulation, social interactions, and affiliative partnerships later in life (Kinreich et al., 2017). The newborn infant is biologically equipped to engage in “bio-behavioral synchrony” (Feldman, 2012) that also contributes to the development of the attachment relationship with their parent(s). Their biosocial development is supported through daily interactive exchanges, as they learn the language (Oller et al., 2001; Kuhl et al., 2006) and emotion display “rules” (Matsumoto and Hwang, 2013) of the culture in which they are growing.

In studies examining behavioral challenges in children, several indicators of parenting are associated with social competence and resilience, as well as early emotional/behavioral disorders. For example, a secure mother–infant attachment not only promotes resiliency, but also protects against such behavioral issues (Letourneau, 1997; Edwards et al., 2006; Cyr et al., 2010; Riva Crugnola et al., 2014). Higher parental cognitive empathy and reflective functioning, both of which measure the capacity to perceive and understand the emotional states of others, are connected to emotion regulation and social competence in children (Wong et al., 2017; Borelli et al., 2020). Furthermore, higher frequency of shared pleasure moments in early childhood between mother and child have been shown to lower the likelihood of emotional/behavioral problems in children and moderate the possible negative effects of parental psychopathology on the development of children’s emotion regulation abilities (Mäntymaa et al., 2015).

Parents who are grossly insensitive to their infant’s affective communication overwhelm their infant’s capacity to develop organized and effective emotion regulation strategies (Lyons-Ruth et al., 1999). Underdeveloped emotion regulation systems often lead to dysregulated emotions and frequently manifest as child internalizing and externalizing behaviors, including excessive or prolonged tantrums, physical aggression, and/or emotional withdrawal and anxiety (Lyons-Ruth, 1996; Calkins et al., 2007; Chambers and Allen, 2007; Beauchaine and Thayer, 2015; Ostlund et al., 2019).

### Maternal Stress and Trauma

An informed approach to understanding the dynamics and developmental implications of early parent–child interactions must also attend to parental wellbeing and psychopathology. The mental health of any primary caregiver can significantly affect early relationships and child development; however, we focus on maternal mental health given the replicated associations between maternal wellbeing and myriad gestational, birth, and caregiving outcomes (Grote et al., 2010; Kingston et al., 2012; Monk et al., 2012; Van den Bergh et al., 2020). A history of adverse experiences in childhood, traumatic stress, and PTSD can impact adult emotion regulation and sensitive and responsive parenting, and in turn impact young children’s social–emotional development.

#### Adverse Childhood Experiences

Adverse Childhood Experiences (ACEs) generally refer to events prior to the age of 18 that may have been distressing or traumatizing for an individual, including psychological, physical, or sexual abuse, physical and emotional neglect, or witnessing interpersonal violence, among other events (Felitti et al., 1998). The original ACEs study (Felitti et al., 1998) demonstrated the significant connection between cumulative exposure to abuse, neglect, or household dysfunction during childhood and multiple adverse health outcomes in adulthood, including increased risk of depression, an array of physical health issues, and early mortality (Chapman et al., 2004; Felitti et al., 2019). In the perinatal period, higher ACE scores are associated with negative pregnancy and birth outcomes, including high-risk pregnancy behaviors (e.g., alcohol and cigarette use), preterm birth, and low birth weight (Chung et al., 2009; Hudziak, 2018; Racine et al., 2018). Pregnancy and the postpartum period are commonly associated with the initial onset or exacerbation of mental health concerns (Munk-Olsen et al., 2016) and women with a history of childhood adversity are disproportionately at risk for clinically significant symptoms of perinatal depression, anxiety, and PTSD (McDonnell and Valentino, 2016; Narayan et al., 2016, 2018; Oh et al., 2016; Meltzer-Brody et al., 2018; Racine et al., 2018; Atzl et al., 2019; Osofsky et al., 2021). In the current study, we therefore expand upon the existing literature to address intergenerational relationships between experiences of adversity in a mother’s own childhood, current maternal PTSD symptoms, and socioemotional and behavioral outcomes for her young child.

#### Maternal Traumatic Stress

Childhood adversity can also lead to cumulative, chronic stress when the event is on-going in duration or uncontrollable in nature, and children do not have a stable or emotionally available caregiver who can support and protect them or help them make sense of the situation(s) (Lieberman and Van Horn, 2009; Slade, 2014). Mothers who faced more adversity in their own childhoods are more likely to use corporal punishment and to display hostile, intrusive, or frightening parenting behaviors (Jacobvitz et al., 2006; Chung et al., 2009). Condon et al. (2019) suggest that mothers who experienced high levels of adversity and stress early in life may have limited lived experiences to draw from in order to offer their children solace from stressful experiences or teach effective coping skills in the presence of current stressors. Intrusive, withdrawing or hostile parenting styles can have direct effects on the infant and young child, as well as contribute to challenges within the mother–child relationship (Lyons-Ruth, 1996). Maternal ACEs in the form of childhood maltreatment are directly correlated with higher levels of maladaptive socioemotional symptoms in infants (McDonnell and Valentino, 2016). There is evidence that maternal childhood trauma leads to disturbed caregiving behavior which in turn interferes with infants developing a secure attachment (Lyons-Ruth and Block, 1996). Furthermore, a mother’s history of ACEs predicts higher behavioral problems and blood pressure levels – a tangible biomarker of stress – in children (Condon et al., 2019). Nevertheless, the impact of maternal childhood adversity and toxic stress on parenting behaviors and child outcomes is probabilistic rather than deterministic. When controlling for concurrent perinatal mental health symptoms, effects of maternal childhood adversity on parenting and child outcomes do not always confer risk (Martinez-Torteya et al., 2014; Muzik et al., 2017; Morelen et al., 2018). However, in toddlers and older children, maternal childhood abuse and adversity more consistently predict risk for emotional and behavioral problems (e.g., Collishaw et al., 2007; Min et al., 2013; Myhre et al., 2014; van de Ven et al., 2020). Thus, a mother’s own childhood adversity and caregiving history may directly impact her child’s development of emotion regulation, often manifesting as behavioral and emotional regulation challenges. This is even more true in the context of maternal post-traumatic stress disorder. For example, maternal PTSD symptoms predict infant emotional reactivity and regulation deficits (Bosquet Enlow et al., 2011), insecure and disorganized attachment (van Ee et al., 2016), dysregulation in preschoolers (Pat-Horenczyk et al., 2015), and internalizing and externalizing problems in children (Schechter et al., 2011; Glaus et al., 2021).

There are multiple psychosocial routes through which maternal PTSD may affect young children. Prenatal PTSD symptoms can affect child development through prenatal exposure to mothers’ disordered stress-response systems and altered epigenetic programming (Glover et al., 2010); however, dysregulated prenatal stress responses are not limited to current stressors and childhood adversity may play a significant role. Bublitz et al. (2014) found that maternal history of severe childhood sexual abuse was associated with increased cortisol awakening response – a biomarker of PTSD – for pregnant women between 25 and 35 weeks’ gestation. In the postpartum period, effects of maternal PTSD on child development may be conferred through disruptions to sensitive and responsive caregiving. Higher severity of PTSD symptoms increase risk for maternal negative representations of the parent–child relationship (Schechter et al., 2005; van Ee et al., 2016). Mothers with PTSD may be emotionally unavailable; experience higher levels of parenting stress; endorse higher levels of aggression toward their children; have increased difficulty reflecting on their child’s needs; have limited empathic responses to their child’s struggles; and display significant difficulties providing sensitive, nurturing care that contributes to security of attachment and healthy child development (Schechter et al., 2008, 2005; Pereira et al., 2012; Muzik et al., 2013; van Ee et al., 2016). Notable child outcomes associated with maternal PTSD and these maladaptive parenting behaviors include difficult temperament, sleep disturbances, and internalizing and externalizing problems, among other challenges (Tees et al., 2010; Hairston et al., 2011; Parfitt et al., 2013; van Ee et al., 2016; Plant et al., 2018). Maternal trauma symptoms have also been found to mediate the relationship between maternal history of childhood trauma/interpersonal violence and child outcomes (Schechter et al., 2011; Fenerci and DePrince, 2018). However, no study to our knowledge has examined the impact of broader maternal ACEs on maternal PTSD symptoms and child behavioral outcomes, a gap the current study will address.

Both past and ongoing stress and trauma can impact parent mental health, parenting behavior, parent perception of the infant, and infant socioemotional development. Infant mental health interventions can support parents with a history of stress and trauma and in turn buffer the negative effects of stress and trauma on infant socioemotional development.

### Infant Mental Health Treatment

The seminal article, “Ghosts in the Nursery” (Fraiberg et al., 1975), spawned the birth of home-based “kitchen table” therapy (Fraiberg et al., 1980) as a preventative intervention model to promote sensitive and nurturing care, reduce maltreatment, and to promote infant wellbeing (Weatherston and Tableman, 2015; Lawler et al., 2017). Fraiberg and colleagues, 1980 documented, with clinical sensitivity and acuity, the myriad of ways a parental history of unresolved childhood loss, separation, abuse, or neglect resulted in the ghosts in the nursery of the traumatized parent, invading the present parent–infant relationship. The very defenses that the parent, when she was a young child, used to ward off the anxiety of uncontained fear (Fraiberg, 1982; Slade, 2014) emerges in parenting in response to the vulnerability of their newborn or young infant. Later termed infant mental health-home visiting (IMH-HV; Tableman and Ludtke, 2020), IMH-HV offers a mix of needs-driven intervention, often combining concrete services, emotional support, developmental guidance, and infant–parent psychotherapy. Weekly, or twice weekly, depending on the needs of the family, the IMH-home visitor meets in the home of the family, with the infant present. The presence of the infant focuses attention on the developing relationship and enables both the parent and the infant to inform the IMH specialist, *via* their interactive exchanges, what is going well and what is challenging. The Michigan Model of IMH-HV is an empirically validated approach to enhancing parenting outcomes (Rosenblum et al., 2020; Julian et al., 2021; Stacks et al., 2021), but research is nascent of its’ impact on the socioemotional wellbeing of the infant. In the presence of a containing, responsive therapist, the parent is helped to grieve unmourned losses, give voice to the fears that went unnoticed in their own childhood, and hear and see the needs of their infant (Malone and Dayton, 2015; Weatherston and Ribaudo, 2020). Infant mental health treatment may help to mitigate the impact of parents’ painful early relationship history on the current parent–child relationship and infants’ wellbeing.

### Current Study

The current study has two distinct aims. The first is to examine the association between maternal ACEs, maternal symptoms of PTSD, and maternal perception of their young child’s socioemotional difficulties. Our second aim is to examine whether participation in IMH-HV improves child socioemotional outcomes and mitigates the associations between maternal PTSD symptoms and child socioemotional and behavioral difficulties. With regard to aim 1, we hypothesize that mothers who report greater exposure to ACEs during their own childhood will report more symptoms of PTSD at study baseline and will report that their young children demonstrate more behavioral and emotional difficulties. Furthermore, we hypothesize that mothers’ PTSD symptoms will mediate the association between maternal ACEs and elevated infant socioemotional and behavioral difficulties. With regard to aim two, we hypothesize that in the context of maternal PTSD symptoms, mothers randomized to IMH-HV treatment will report their toddlers display more positive socioemotional functioning compared to toddlers randomized to the control condition.

## Materials and Methods

### Methods

Participants were 58 mother–infant/toddler dyads who completed baseline and 12-month follow up visits as part of a larger longitudinal randomized control trial (*N* = 73) testing the efficacy of the relationship-based IMH-HV treatment model. Families were eligible based on child age (<24 months at enrollment) and endorsement of at least two of the following: eligibility for public assistance, probable maternal depression, perceived parenting challenges, or history of maternal childhood adversity (ACE score >3). Eligible parents were >18 years of age, had legal custody of their child at enrollment, and did not endorse symptoms of substance use disorders or psychosis. Diagnoses such as depression, anxiety, attention deficit hyperactivity disorder, etc., were *not* grounds for exclusion. The study was reviewed and approved by the University of Michigan Institution Review Board. The participants provided their written informed consent to participate in this study.

This particular study included dyads whose children were born at the time of study enrollment and completed 12-month follow-up visits. Seven families were enrolled in the study during pregnancy, resulting in no child baseline data. Eight additional families were lost to follow up. Aside from child age, there were not demographic differences between those who were retained in the study and those who were not, suggesting there was no differential attrition. Given the small sample size, it was not possible to use intent-to-treat analyses. Instead, only those participants with data at the 12 month follow-up were included. Families who did not complete later follow-up visits were evenly split across the treatment and control conditions (4 from each). Additionally, some families completed later follow-up evaluation visits even after they ended participation in the intervention, which ended for some families before 12 months largely due meeting mutually agreed upon goals. Further details can be found in Riggs et al. (2021).

At the time of study enrollment, average maternal age was 32.5 years (SD = 5.41) and the average age of the child was 11.9 months (SD = 6.57). The majority of the sample identified as a racial or ethnic minority (62.1%); White mothers comprised 37.9% of the sample. Nearly 40% of the families (*n* = 22) reported household incomes of less than $40,000 and the average number of ACEs (Felitti et al., 1998) experienced by mothers was 3.64 (SD = 2.40) out of a possible 10, significantly above the national mean ACE score of 1 (Merrick et al., 2018). See Table 1 for additional demographic characteristics.

**TABLE 1:** Frequencies (and percentages) for social demographic characteristics (*N* = 58) at baseline assessment.

	*n* (%)	*M*	SD
**Participant demographics**			
Mother age		32.65	5.23
Child age in months		11.95	6.19
**Household income range**			
0–$19,999	9 (15.8)		
20,000–$39,999	13 (22.4)		
40,000–$59,999	13 (22.4)		
60, 000–$79, 999	5 (8.5)		
80,000 and above	17 (29.2)		
**Race/ethnicity**			
White	22 (37.9)		
Racial or ethnic minority	36 (62.1)		
**Education level**			
High school diploma or less	8 (13.8)		
Some college or associates degree	12 (20.7)		
College or Voc. Tech degree	22 (37.9)		
Postgraduate degree	16 (27.6)		
**Marital status**			
Currently married	47 (81.0)		
Not currently married	11 (19.0)		

*One participant did not wish to report income data for her family.*

### Measures

#### Maternal Adverse Childhood Experiences

The ACEs questionnaire is a 10-point measure used to assess harmful events (i.e., abuse, neglect, or household dysfunction) that occurred prior to the age of 18. The 10 events include psychological abuse, physical abuse, sexual abuse, physical neglect, emotional neglect, parental divorce, family member mental illness, substance abuse by a family member, incarceration of a family member, and domestic violence. At baseline data collection, participants endorsed (yes) or denied (no) exposure to the 10 items. A total score was calculated by summing the endorsed items, with higher total scores indicating a greater number of adverse events. See Table 2 for a summary of descriptive statistics for the ACE questionnaire.

**TABLE 2:** Descriptive statistics for key study variables.

	Baseline			12-month follow-up			
Variable	*M*	SD	Range	*M*	*M*	Range	Possible range
**Maternal measures**							
ACE score	3.64	2.4	0–10	–	–		0–10
PTSD score (PCL-5)	22.89	17.42	1–67	14.06	13.73	0–69	0–80
**Child measures**							
BITSEA Problems score	–	–	–	11.14	5.92	2–27	0–62
DECA Attachment score	50.94	8.54	33–67	51.94	9.86	33–66	
DECA Initiative score	51.72	8.63	33–72	52.35	10.04	32–72	

*ACE, Adverse Childhood Experiences questionnaire; PCL-5, PTSD Checklist for DSM-5; BITSEA, Brief Infant-Toddler Social and Emotional Assessment; DECA, Devereux Early Childhood Assessment.*

#### Maternal Posttraumatic Stress Disorder Symptoms

The PTSD Checklist for DSM-5 (PCL-5; Weathers et al., 2013; Blevins et al., 2015) is a 20-point self-report measure that assesses symptoms consistent with PTSD diagnostic criteria. Respondents are asked to report on symptoms within the past month, for example, how much they have been bothered by “*Repeated, disturbing, and unwanted memories of the stressful experience?*” or “*Feeling distant or cut off from other people?*” The PCL-5 is scored on a 0–4 Likert scale, increasing in severity from 0 (*not at all*) to 4 (*extremely*), with total scores ranging from 0 to 80. This measure can be used as a screener for a provisional diagnosis of PTSD, wherein the suggested clinical cut-off for total scores falls between 31 and 33. In this study, a total score was calculated by summing the individual items, with higher scores reflecting greater severity of symptoms. Prior reports indicate sound psychometric properties of the PCL-5 (Blevins et al., 2015), and reliability was high in the current sample (α = 0.94). PTSD symptoms data was collected at baseline and 12 months.

#### Infant-Toddler Socioemotional Development

The Brief Infant-Toddler Social Emotional Assessment (BITSEA; Briggs-Gowan et al., 2004) is a 44-item parent-report measure for children ages 12–35 months. It is a screener used to assess social, emotional, behavioral problems, and delays in competence as well as to identify children measuring at-risk in multiple areas. Parents rate each descriptive comment about their child (e.g., “My child often gets very upset”) from 0 (*not true/rarely*) to 2 (*very true/often*). In this study, the 31-item total Problem Score was utilized, with a possible range of scores from 0 to 62. Higher Problem Scores indicate greater difficulties with child internalizing, externalizing, or regulation capacities. The BITSEA can only be administered after the infant is 12 months old. Because a number of families began treatment prior to the child’s first birthday, there was too much missing data at baseline to use in analyses. As such, the BITSEA is only used in study aim 1 to assess elevated social emotional difficulties at 12-months.

#### The Devereux Early Childhood Assessment

Because the BITSEA is only administered after the infant is 12 months old, we also included the Devereux Early Childhood Assessment-Infant (DECA-I) and the Devereux Early Childhood Assessment-Toddler (DECA-T; Powell et al., 2007) to enable a large enough sample size to accommodate the planned analyses. The DECA-I and DECA-T are standardized parent-report measures of children’s emotional and behavioral adjustment. The measure generally assesses positive and protective factors demonstrated by resilient infants and toddlers. There are two subscales that are consistent across both versions of the Devereux Early Childhood Assessment (DECA); the Initiative scale, which measures behaviors used by the infant/toddler to meet their needs, and the Attachment/Relationships scale, which assesses social and emotional behavior and regulation exhibited between the infant/toddler and their caregiver. Example items on the Initiative scale include “…*how often did the child try to do things for herself/himself?*” and “…*how often did the child try to comfort others?*” Example items on the Attachment/Relationships scale include “…*how often did the child seek comfort from familiar adults?*” and “… *how often did the child express a variety of emotions (e.g., happy, sad, mad)?*”

For the infant version of the assessment (DECA-I; used for children 0–18 months of age), there are 18 items on the Initiative subscale and 15 items on the Attachment/Relationships subscale. For the toddler version of the assessment (DECA-T; used for children 18–36 months of age), there are 11 items on the Initiative scale and 18 items on the Attachment/Relationships scale. Scoring on both forms of the DECA yield standardized *T*-scores, where scores greater than 60 indicate a strength, scores less than 40 indicate an area of concern, and scores between 40 and 60 indicate typical socioemotional development. Subscale reliability for both versions of the DECA were acceptable (Cronbach’s alpha for the Initiation subscale ranged from 0.68 to 0.85; Cronbach’s alpha for the Attachment/Relationships subscale ranged from 0.90 to 0.92).

### Analytic Strategy

Data were analyzed using IBM SPSS for Windows, Version 26. Frequencies and percentages were calculated for all categorical variables. Means and standard deviations were calculated for the continuous variables. Before proceeding with the primary study analysis, data were screened for errors and extreme values to ensure that data met assumptions for statistical analysis and to understand factors that may affect interpretation of the findings. Variables were checked for outliers and assessed for normal distribution of differences in the scores; all assumptions for the planned analyses were met.

For our first aim, we conducted a multiple regression mediation model using PROCESS (Hayes, 2013) to explore the relationship between the mothers’ baseline experience of childhood adversity, the severity of maternal PTSD symptoms, and infant/toddlers’ socioemotional and behavioral outcomes at the 12 month follow up wave. We used the BITSEA Problem score as the measure of socioemotional and behavioral outcomes for this aim, given its wide use as a measure of child socioemotional functioning and excellent psychometric properties.

For our second aim, we conducted a multiple regression moderation model (Hayes, 2013) to explore the impact of treatment condition on infant/toddler socioemotional outcomes and the potential role of maternal PTSD symptoms in this effect. For this aim, we utilized the DECA subscales as our measures of child socioemotional development. This was necessary in order to control for baseline socioemotional development because the DECA can be administered to infants less than 12 months of age, while the BITSEA can only be administered once the child is older than 1 year. Given that many of the families began treatment when their child was less than 1 year, there was not enough BITSEA data available to use to examine pre/post treatment efficacy. DECA Initiation and Attachment subscales were evaluated separately.

## Results

### Preliminary Analyses

Prior to conducting primary analyses, we examined descriptive and correlational data among our study variables of interest. See Tables 2, 3 for additional detail. Overall, maternal experiences of childhood adversity were relatively high compared to population estimates, and ACE scores ranged from 0 to 10 (*M* = 3.64; SD = 2.40). Although average scores were below the cutoff used to identify probable PTSD diagnoses (suggested cutoff of 33), there was some variability in the sample, with a number of participants endorsing PTSD symptoms scores above the clinical cutoff (*n* = 17; 23.30% of sample). Maternal PTSD symptom scores reported at the 12-month data collection visit were relatively lower but included a number of participants (*n* = 8; 12.1% of sample) whose 12-month assessment of PTSD symptoms indicated probable diagnosis of PTSD.

**TABLE 3:** Correlations among key study variables.

Variable	ACE score	PCL-5 baseline	PCL-5 12-month	BITSEA Problems baseline	BITSEA Problems 12-months	DECA Attachment baseline	DECA Attachment 12 months	DECA Initiative baseline
**Parent measures**								
ACE score (baseline)	–							
PCL-5 (baseline)	0.42***	–						
PCL-5 (12-month)	0.15^c^	0.44***	–					
**Child measures**								
BITSEA Problems (baseline)	0.39*	0.10^e^	−0.05^f^	–				
BITSEA Problems (12-month)	0.36**	0.45***	0.22^a^	0.53***	–			
DECA Attachment (baseline)	−0.02^f^	0.05^e^	−0.01^f^	−0.56***	0.01^f^	–		
DECA Attachment (12-month)	−0.11^d^	−0.13^c^	0.06^e^	−0.43*	−0.52**	0.34**	–	
DECA Initiative (baseline)	0.08^e^	0.24^a^	0.08^e^	−0.36*	0.05^e^	0.69***	0.15^c^	–
DECA Initiative (12-month)	−0.19^b^	−0.14^c^	0.0^f^	−0.42*	−0.37**	0.35**	0.68***	0.30*

*p < 0.05; **p < 0.01; ***p < 0.001.

*^a^p ranges from 0.051 to −0.10; ^b^p ranges from 0.101 to 0.20; ^c^p ranges from 0.201 to 0.30; ^d^p ranges from 0.301 to 0.50; ^e^p ranges from 0.501 to 0.70; ^f^p > 0.701. ACE, Adverse Childhood Experiences questionnaire; PCL-5, PTSD Checklist for DSM-5; BITSEA, Brief Infant-Toddler Social and Emotional Assessment; DECA, Devereux Early Childhood Assessment.*

Over a quarter of the sample of toddlers were rated above the socioemotional problems cutoff score at both data collection time points (*n* = 20; 27.4% of sample), representing a subgroup of children with high levels of socioemotional problem behavior. Child protective and positive socioemotional factors were also examined using the DECA. On the Attachment subscale of the DECA, scores indicated that most children were in the “typical” range at baseline (*M* = 50.94; SD = 5.92) and 12-month (*M* = 51.94; SD = 9.86) data collection visits. Some parents rated their children as having “needs” (baseline and 12-month follow up *n* = 7; 9.6%) or “strengths” (baseline *n* = 10; 13.7%; 12-month follow up *n* = 17; 23.3%) on the Attachment subscale. Likewise, when evaluating the Initiative subscale of the DECA, scores indicated that most children in this study were in the “typical” range at baseline (*M* = 51.72; SD = 8.63) and 12-month data collection visits (*M* = 52.35; SD = 10.04), with some children rated as having needs (baseline *n* = 4; 5.5%; 12-month follow up *n* = 5; 6.8%) or strengths (baseline *n* = 9; 12.3%; 12-month follow-up *n* = 17; 23.3%) in this area.

Examination of bivariate correlations among study variables revealed several significant associations among key study variables. See Table 3 for additional information.

### Primary Analyses

**FIGURE 1:**
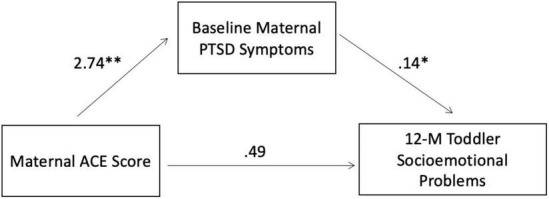
Regression analysis summary for parent variables predicting toddlers’ socioemotional problems. *R*^2^ = 0.25 (*N* = 58, *p* = 0.0004). *p = 0.0041; ^**^p = 0.0013.

Consistent with our first study aim, we examined the association between maternal ACEs, maternal symptoms of PTSD, and toddler socioemotional difficulties. We hypothesized that mothers who reported greater exposure to childhood adversity would report greater symptoms of PTSD (H1). As shown in Table 3, this hypothesis was supported. There was a moderate, positive bivariate association between maternal ACE score and baseline maternal PCL-5 score (*r* = 0.42, *p* = 0.000), wherein mothers who reported greater experiences of childhood adversity reported greater symptoms of PTSD when they entered our study. We also hypothesized that toddlers of mothers with greater experiences of childhood adversity would demonstrate more behavioral and emotional difficulties (H1). This relationship was also supported; as shown in Table 3, there was a moderate, positive association between maternal ACE score and BITSEA Problem score at baseline (*r* = 0.39, *p* = 0.02) and 12-months (*r* = 0.36, *p* = 0.006). This first hypothesis was also supported within the multiple regression models (see Figure 1).

Our second hypothesis was that PTSD symptoms would mediate the association between maternal exposure to childhood adversity and increased risk of child socioemotional and behavioral difficulties in the next generation (H2). Prior to mediation analyses, maternal ACE scores were positively associated with maternal PTSD symptoms measured at baseline and toddlers’ socioemotional difficulties as measured by the BITSEA at the 12-month follow-up, such that mothers with more ACEs had greater PTSD symptoms and reported more behavioral and emotional difficulties among their toddlers. Mediational analyses in PROCESS (Hayes, 2013) revealed a significant mediation model [(*F*(2,55) = 9.2, *R*^2^ = 0.25, *p* = 0.0004], see Figure 1. After controlling for the effect of the proposed mediator – baseline maternal PTSD symptoms – the direct effect of ACEs on child emotional problems was no longer significant (*B* = 0.20, *t* = 1.57, *p* = 0.123), and there was an indirect effect of maternal ACEs on the BITSEA Problem score through maternal PTSD symptoms (β = 0.16, bootstrapped 95% CI = 0.07–0.71). Thus, having a higher number of ACEs predicted more PTSD symptoms among mothers, which in turn predicted more child problems.

Our second aim was to examine how participation in an in-home, relationally based intervention impacts child socioemotional development and whether it mitigates the association between maternal PTSD symptoms and child outcomes.

**FIGURE 2:**
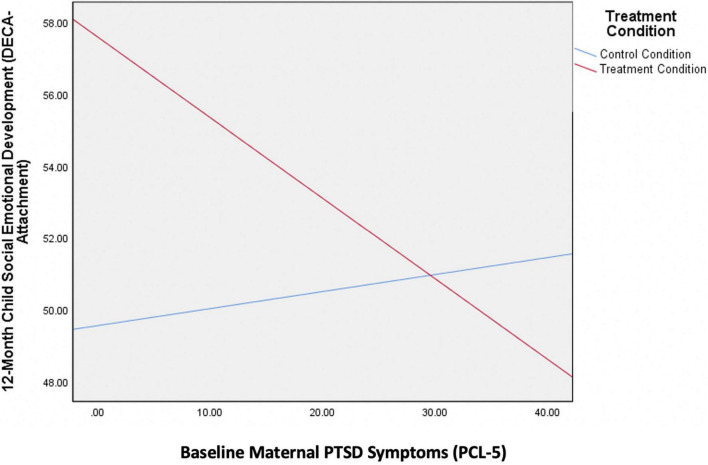
Interaction between maternal PTSD symptom at baseline and treatment condition on child socioemotional development.

As seen in Table 4, the overall moderation model estimating the 12-month DECA Attachment scale was significant [*R*^2^ = 0.20, *F*(4,53) = 3.23, *p* = 0.02]. Baseline ratings on the DECA Attachment subscale predicted 12-month ratings on the DECA Attachment subscale (*t* = 2.82, *p* = 0.006). There was a trend-level main effect of treatment on DECA Attachment scores (*t* = 1.93, *p* = 0.06) wherein mothers randomized to the treatment condition rated their toddlers higher on this scale while controlling for baseline levels. This implies that in the full sample, treatment was marginally effective at improving toddler socioemotional outcomes. There was also a significant interaction between treatment condition and maternal PTSD symptoms at baseline (*t* = −1.76, *p* = 0.04). Specifically, mothers randomized to receive IMH-HV treatment who had lower PTSD symptoms at the start of treatment rated their toddlers higher on the DECA Attachment scale compared to mothers with subclinical baseline PTSD symptoms who did not receive treatment. Thus, IMH-HV treatment was most associated with positive child socioemotional development when mothers entered treatment with fewer PTSD symptoms, see Figure 2. When mothers entered the study with higher PTSD symptoms suggestive of a probable diagnosis of PTSD, their toddlers had similar scores on the DECA Attachment subscale regardless of treatment condition.

**TABLE 4:** Main and interaction effects of maternal PTSD symptoms and intervention on DECA Attachment and Initiative subscales.

Variables	*b*	SE	*t*	*p*
**Model 1**	**Child social emotional development – 12-month DECA Attachment**			

Constant	28.53	8.02	–	
Treatment	8.03	4.16	1.93	0.06
Maternal baseline PTSD score	0.05	0.11	0.42	0.67
Treatment by baseline PTSD symptoms (moderator)	−0.27	0.15	−1.76	0.04
Control variables				
Child socioemotional development (baseline)	0.41	0.15	2.82	0.006

**Model 2**	**Child social emotional development – 12-month DECA Initiative**			

Constant	33.85	8.04	–	
Treatment	−0.55	4.09	−0.13	0.89
Maternal baseline PTSD score	−0.21	0.11	−1.91	0.06
Treatment by baseline PTSD symptoms (moderator)	0.08	0.15	0.50	0.62
Control variables				
Child socioemotional development (baseline)	0.42	0.15	2.72	0.009

*Model 1: R^2^ = 0.20, F(4,53) = 3.23, p = 0.02.*

*Model 2: R^2^ = 0.18, F(4,50) = 2.73, p = 0.04.*

*Maternal PTSD symptoms measured by the PTSD Checklist for DSM-5 (PCL-5); Child socioemotional development measured by the Devereux Early Childhood Assessment-Infant (DECA-I) and the Devereux Early Childhood Assessment-Toddler (DECA-T).*

As shown in the lower portion of Table 4, the overall moderation model estimating the 12-month DECA Initiative scores was also significant [*R*^2^ = 0.18, *F*(4,50) = 2.73, *p* = 0.04]. Baseline ratings on the DECA Initiative subscale predicted 12-month ratings on the DECA Initiative subscale (*t* = 2.72, *p* = 0.009). Treatment condition was not associated with 12-month rating on the DECA Initiative subscale (*t* = −0.13, *p* = 0.89). Baseline maternal PTSD symptoms were a trend-level predictor of DECA Initiative ratings when accounting for the other terms in the model.

There was no significant interaction between treatment condition and baseline PTSD symptoms in the model predicting DECA Initiative scores (*t* = 0.50, *p* = 0.62).

## Discussion

The current study had two aims. First, we aimed to determine if maternal PTSD symptoms mediate the relation between maternal history of adversity and child outcomes. Second, we aimed to test whether IMH-HV was efficacious in improving child outcomes, particularly in the context of maternal PTSD symptoms. Each result will be discussed below.

### Maternal Adverse Childhood Experiences, Posttraumatic Stress Disorder, and Child Outcomes

Our study provides further support for the notion that childhood adversity may increase the likelihood of maternal mental health challenges and thus negative consequences for their offspring. We interpret these associations to be probabilistic, and not deterministic, given the findings that the impact of adversity is mediated by maternal PTSD symptoms, which are amenable to psychotherapeutic intervention. Current symptoms of maternal PTSD were observed to mediate the influence of maternal childhood adversity on infant socioemotional wellbeing suggesting that prenatal screening for maternal symptoms of PTSD is warranted (Erickson et al., 2019). Treating maternal PTSD symptoms sooner rather than later may reduce maternal suffering. Such amelioration affords the developing relationship between mother and infant the opportunity to unfold unhindered by extremely insensitive, misattuned maternal behaviors, such as dissociation, avoidance, or flooding, which can disrupt the sensitive caregiving and the affective communication between parent and infant (Lyons-Ruth et al., 1999).

Importantly, young children presenting with externalizing and internalizing disorders may be adapting to parental behavior in order to sustain the attachment relationship, even if costly to their psychosocial development (Sroufe, 2009; Borelli et al., 2020). Mothers who are experiencing intrusive thoughts while interacting with their infant may demonstrate behaviors that are disruptive to the infants’ affective communication, such as dissociation, aversive hostility, or withdrawal (Lyons-Ruth et al., 1999). Infants of mothers who are struggling with PTSD must adapt to caregiving that is more likely to be negative, intrusive, or in other ways persistently insensitive or alarming (Lyons-Ruth, 1996; Schechter et al., 2014). As Fraiberg noted long ago, infants who are routinely unprotected from overwhelming affects and frequently left in a state of helpless despair develop psychological defenses against the aversiveness of interaction with the parent (Fraiberg, 1982; Weatherston and Ribaudo, 2020). The substrate of socioemotional health is laid in the first months of life; when the infant experiences frequent lapses in parental responsivity, or when the parent becomes the source of alarm (Hesse and Main, 2000) the infant is more likely to develop defensive strategies such as avoidance or aggression (Fraiberg, 1982) that result in an increased risk of child psychopathology (Lyons-Ruth, 1996; Plant et al., 2018). The data suggests that practitioners who are consulted due to concerns regarding young children with socioemotional problems should pay careful attention to assessing parental mental health, including assessment of subclinical levels of PTSD. Treatment of children’s behavior problems in the absence of treating parental mental health may be ineffective. In addition, the association between parental psychopathology and childhood socioemotional problems lends evidence to the need for family systems and dyadic assessment (Shonkoff et al., 2012; Erickson et al., 2019). For instance, psychotherapists providing individual treatment for adults who are parents of young children can promote two generation approaches by encouraging their patients who have a history of early adversity or depression to access IMH-HV services, in addition to their continuing their own psychotherapy. However, it is noted that such an approach would require significant coordination and teamwork, ideally within a reflective consultation/supervision group with the therapists to reduce the risk splitting/fragmentation and confusion for the traumatized parent(s) (Biggart et al., 2017; Green, 2018).

### Efficacy of Infant Mental Health-Home Visiting

Building upon previous reports that IMH-HV is an effective intervention that promotes maternal sensitivity, responsivity, and reflective functioning (Rosenblum et al., 2020; Julian et al., 2021; Stacks et al., 2021), the current study explored the effectiveness of IMH-HV for improving child outcomes among mothers with a history of childhood adversity and current PTSD symptoms. Results indicated positive socioemotional outcomes for children of mothers who received IMH-HV compared to the control group, with the best outcomes seen for children of mothers with low to moderate PTSD symptoms who received the intervention. Attention to early intervention and the emerging relationship (Fitzgerald et al., 2011), before early childhood adaptations become traits (Perry et al., 1995), is a hallmark of infant mental health work. Our findings suggest that dyadic intervention for mothers with low to moderate PTSD symptoms helps promote child socioemotional wellbeing. Mothers with low to moderate PTSD symptoms may respond more quickly to IMH-HV intervention than mothers with more severe symptomology. IMH-HV services are designed to provide intervention until the infant’s third birthday. This study only examined child wellbeing after 12 months of service. A longer duration of treatment of both individual and dyadic psychotherapy may be warranted for mothers with more PTSD symptomology to effect improvement in children’s socioemotional functioning. Some mothers may need a sustained period of intervention to stabilize their symptoms (Stacks et al., 2021). They may need time to experience “being held in the mind of another” (O’Rourke, 2011, p. 170) and understanding their own mental states (Suchman et al., 2018) before they can regulate their own emotions well enough to help their child co-regulate. For mothers with history of relational harm, the “felt sense” of being cared for that fuels mothering may be thin. The therapeutic relationship serves as a corrective emotional experience, but that takes time to develop, especially for mothers with a history of early childhood adversity (Stacks et al., 2021). In addition, once the mother begins to understand her mental states, it will still take time for the child to shift their “expectation” of interactions. The longer they have adapted to a particular attachment template, the longer it may take to develop the confidence in using the parent as a source of comfort (Stovall-McClough and Dozier, 2004; Barlow et al., 2021). It is also possible that children of mothers with severe PTSD symptoms did benefit from the intervention, but that these mothers were not reliable reporters of their infants’ symptoms early on and became more insightful as treatment progressed. This reporter-bias would have masked treatment effects in this group. Further research is necessary to examine the efficacy of the model, including the duration and intensity required, to effect dyadic change in the highest risk group.

Importantly, many of the mothers faced systemic inequity and oppression, in addition to interpersonal harm, which add to the complexity and severity of traumatic stress (Cyr et al., 2010). The challenges of developing a therapeutic alliance with traumatized individuals can be exacerbated when there are obvious differences in social identities, especially when clinicians are uncomfortable addressing racial and ethnic differences (Owen et al., 2017). Further research is necessary to examine the contribution of client and therapist match to therapeutic outcomes.

### Contributions and Limitations

Our study has several strengths, including randomization and a control group, low attrition, a racially and economically diverse sample, and longitudinal data. It expands and refines attention to the mounting load of PTSD symptoms on the mother and infants’ wellbeing. It provides evidence that IMH-HV is efficacious at improving child socioemotional outcomes and can be especially effective for dyads where the mother has low to moderate PTSD symptoms. However, for mothers with more PTSD symptoms, it’s possible that longer durations of treatment may be more effective at reducing mother’s dysregulation before the positive impact on the infant is observable.

A limitation of the study is that we relied on self-report of PTSD symptoms, rather than a clinical interview. Because avoidance is a central feature of PTSD, it may be that this led to under-reporting of PTSD symptoms. If this is true, it could impact the results of this study, especially considering that IMH-HV was found to be especially effective for mothers with low to moderate PTSD symptoms. If self-reported PTSD symptom rates were lower due to participant avoidance, it may be that the intervention is effective for those with greater PTSD symptoms than we initially thought. Similarly, we did not ask the mothers to elaborate which traumatic experience they were reflecting upon while answering the PCL-5 questions. Knowing the nature of the traumatic event(s) that contribute to dyadic disruption may offer the capacity for more refined treatment. For instance, there are likely to be qualitative differences in interactions with a baby if parent is reporting PTSD symptoms related to a recent incident of interpersonal violence as opposed to recalling physical abuse in their childhood (Schechter et al., 2014).

Another limitation of the study is the reliance on parental self-report for measures of infant wellbeing. Mothers who are coping with multiple stressors, including PTSD, may have a negative bias which contributes to perceiving the infant as difficult (Romero et al., 2021) or may present as avoidant of their infant’s challenges. Future research would benefit from having both parent-reported and observer-rated measures of infant socioemotional development.

Additionally, the gold standard for treatment studies is intent-to-treat analyses, which were not possible in this trial. Thus, the attrition rate may affect the generalizability of the findings. Finally, there is some evidence of the utility of the DECA in samples of preschool children from low-income backgrounds, though the behavioral concerns subscale may not adequately assess culturally and linguistically diverse children in low-income families (Bulotsky-Shearer et al., 2013). Thus, this measure may not have captured the full range of socioemotional concerns or strengths of our population.

### Future Directions

Our sample included many women who have faced systemic inequalities and oppression. Experiences with systems of care guided by dominant culture may negatively impact the epistemic trust (Fonagy and Allison, 2014) that contributes to the development of a therapeutic relationship. Infant mental health therapists, though engaged in reflective practice, may unwittingly interfere with the development of a trusting relationship by engaging in microaggressions or being culturally insensitive. Mothers whose history of relational harm intersects with societal harm, may present with co-morbid and severe traumatic life-events (e.g., life-threatening encounter with law enforcement) and thus demonstrate related mental health challenges. They may require longer, more intensive or multi-modal, and interdisciplinary interventions that are culturally responsive and humble, while also recognizing often unacknowledged sources of resiliency and support (Parker, 2021).

A majority of our sample (70%) was married. This study did not specifically address how paternal support contributes to the infant’s mental health. Given the influence of the father–infant relationship on infant mental health, *via* the marital relationship and direct interaction with the infant, future studies could reveal the impact of the marital relationship on infant socioemotional wellbeing (Hall et al., 2014; Korja et al., 2016).

In conclusion, this study contributes to the body of research that suggests that maternal mental health influences the socioemotional wellbeing of young children. It provides evidence that IMH-HV is effective in supporting the socioemotional health of infants and young children of mothers, especially for those who experience low to moderate PTSD symptoms. It suggests that a longer period of intervention may be required to impact the mother’s capacity to support their infant’s socioemotional wellbeing when she struggles with more severe PTSD symptomology.

## Data Availability Statement

The raw data supporting the conclusions of this article will be made available by the authors, without undue reservation.

## Ethics Statement

The studies involving human participants were reviewed and approved by the University of Michigan Institutional Review Board. Written informed consent to participate in this study was provided by the participants’ legal guardian/next of kin.

## Author Contributions

MM and KR obtained funding and oversaw all aspects of the clinical trial. JuR, JL, and JeR conceptualized and designed the study for this manuscript. JJ organized the database. JuR, JL, JeR, and JJ performed the statistical analyses. JuR wrote the first draft of the manuscript. JuR, JeR, NE, and JL wrote sections of the manuscript. All authors contributed to manuscript revision, and read and approved the submitted version.

## Conflict of Interest

The authors declare that the research was conducted in the absence of any commercial or financial relationships that could be construed as a potential conflict of interest.

## Publisher’s Note

All claims expressed in this article are solely those of the authors and do not necessarily represent those of their affiliated organizations, or those of the publisher, the editors and the reviewers. Any product that may be evaluated in this article, or claim that may be made by its manufacturer, is not guaranteed or endorsed by the publisher.
